# Continuous bed motion versus step-and-shoot acquisition in LAFOV PET/CT: insights from multi-phantom and patient studies

**DOI:** 10.1186/s40658-026-00867-3

**Published:** 2026-04-12

**Authors:** P. M. Linder, W. Lan, E. Calderón, I. Rausch, C. la Fougère, F. P. Schmidt

**Affiliations:** 1 Department of Nuclear Medicine and Clinical Molecular Imaging, University hospital Tuebingen, Tuebingen, Germany; 2 QIMP Team, Center for Medical Physics and Biomedical Engineering, Medical University of Vienna, Waehringer Guertel 18–20/4L, 1090 Vienna, Austria; 3 Cluster of Excellence iFIT (EXC 2180) “Image Guided and Functionally Instructed Tumor Therapies”, University of Tuebingen, Tuebingen, Germany; 4 Werner Siemens Imaging Center, Department of Preclinical Imaging and Radiopharmacy, Eberhard Karls University Tuebingen, Roentgenweg 13, 72076 Tuebingen, Germany

**Keywords:** LAFOV PET, Total-body PET, Continuous-bed-motion scan, CBM, Step-and-shoot, Quantification

## Abstract

**Background:**

Continuous bed motion (CBM) allows flexible extension of the scan range compared to conventional step‑and‑shoot (S&S) acquisition but has not yet been evaluated in long axial field‑of‑view (LAFOV) PET/CT. This study systematically assessed the impact of CBM on image quality, noise, and quantitative performance in the Biograph Vision Quadra LAFOV PET/CT using multi‑phantom and patient scans compared to S&S.

**Methods:**

A uniform tube phantom and a NEMA IEC phantom, positioned centrally and off-centre, were scanned across bed speeds (2.8–50 mm/s), sensitivity modes and scan ranges (106 and 150 cm) to evaluate image uniformity, axial count profiles, noise and contrast recovery coefficients (CRC). Ten oncological patients receiving [^18^F]PSMA-1007 or [^18^F]FDG underwent sequential CBM (2.8 mm/s, 378 s) and S&S (300 s) scans. Image noise, net true counts, and liver and lesion SUV values were compared using paired statistics and Bland–Altman analysis, along with PSMA expression scores.

**Results:**

For comparable count statistics and image noise, CBM required a prolonged acquisition (378 s) to match the S&S (300 s) protocol, resulting in comparable image quality for phantoms and patients. CRC and image uniformity were preserved across all evaluated conditions, even at the FOV’s axial edge (50.5 cm) for 8.4 mm/s (22 mm sphere: CRC 76% S&S vs. 71% CBM). In patient scans, minor differences in axial count profiles, net true counts, and SUV values (SUV_mean_ bias − 0.1 (liver) and − 0.8 (lesions)) did not affect clinical scores.

**Conclusions:**

The prolonged CBM protocol provides image quality and quantitative performance comparable to S&S in LAFOV PET/CT. While the reconstructed image range remains constrained by CT coverage, the patient scan comparison with 106 cm scan range, together with extended range phantom measurements, indicate that CBM can support scan range extension beyond 106 cm without compromising diagnostic accuracy.

**Supplementary Information:**

The online version contains supplementary material available at 10.1186/s40658-026-00867-3.

## Background

The introduction of long axial field-of-view (LAFOV) PET/CT scanners has transformed total-body PET imaging by substantially increased sensitivity and axial coverage [1], enabling simultaneous imaging of all major organs within a single bed position and reduced scan times and lower tracer doses [2–4]. Moreover, LAFOV systems expand clinical and research applications in settings with low count statistics, such as in immuno-PET [5, 6], Y-90 imaging and dosimetry [7, 8], paediatric imaging [9, 10], dual-tracer imaging [11, 12] and facilitate dynamic total-body acquisitions, including parametric [13, 14] and perfusion studies [15, 16].

While a scanner with full head-to-toe axial coverage, such as the uEXPLORER with its 198 cm axial field of view (aFOV) [17], is appealing for single bed position total-body imaging, the majority of LAFOV PET/CT systems adopt a deliberate compromise. With aFOVs typically ranging from 106 to 150 cm [18–20], these systems offer a balanced trade-off between costs, installation complexity, and the limited benefit of highly oblique lines of response (LORs). While this head-to-thigh coverage is sufficient for most of the clinical and research applications, certain clinical scenarios require full-body imaging. These can include patients with melanoma [21, 22], lymphoma [23], neurofibromatosis type 1 [24], fever of unknown origin, systemic inflammatory diseases [25, 26] and rheumatologic conditions such as arthritis [27]. Full-body imaging is particularly important in cases involving diffuse bone metastases or distant lesions, such as seen in advanced prostate and breast cancer [28, 29], to ensure comprehensive lesion detection and accurate assessment of disease extent. In standard axial field-of-view (SAFOV) scanners, to extend the image range beyond the physical aFOV, typically sequential scans with multiple overlapping bed positions are employed. In LAFOV scanners, however, where a single bed position already provides substantial axial coverage, this discrete step-and-shoot (S&S) approach becomes increasingly inefficient. With the conventionally used 50% overlap, S&S lacks flexibility, allowing only coarse increments in image range (e.g., 106 cm, 159 cm, and 212 cm for one, two, and three bed positions, respectively), preventing efficient coverage of intermediate ranges tailored to individual clinical needs.

A more flexible alternative is continuous bed motion (CBM) [30], in which the patient bed is translated smoothly through the scanner during acquisition, enabling seamless coverage across arbitrarily extended axial ranges. In SAFOV PET scanners, studies have demonstrated comparable image quality between CBM and S&S modes [31, 32], along with additional benefits of CBM in terms of operational efficiency and patient comfort [32–34]. Implementing CBM in LAFOV PET scanners introduces unique challenges. To maintain the advantage of short acquisition times provided by LAFOV systems, appropriately high bed speeds must also be employed in CBM. For largely extended scan ranges, such as in patients with melanoma involving the lower extremities, even higher speeds may be applied in these regions to keep total acquisition times reasonable short. This demands careful consideration to ensure that increased and varying bed speeds do not compromise quantitative accuracy or image uniformity. Whereas in SAFOV PET systems the bed typically translates the entire aFOV during acquisition, LAFOV PET systems may require only limited bed movement relative to their longer aFOV. In the case of the Biograph Vision Quadra (Siemens Healthineers, Knoxville, TN, USA), the LAFOV PET/CT scanner evaluated in this study, the sensitivity profile also depends on the selected sensitivity mode. While the ultra-high sensitivity (UHS) mode yields a typical triangular sensitivity profile, the high sensitivity (HS) mode results in a flatter, more uniform trapezoidal profile and noise distribution [18, 35]. The combined effect of relatively short bed translation and different sensitivity profiles has not yet been systematically investigated in the context of LAFOV PET systems.

To address these gaps, we performed a comprehensive evaluation of the CBM performance for the Biograph Vision Quadra using both phantom and patient scans compared against stationary single-bed acquisitions as the reference. In conventional SAFOV PET scanners, S&S typically denotes multiple bed positions, which is not supported in the LAFOV scanner Biograph Vision Quadra; in the context of LAFOV PET/CT systems, we therefore propose the term single-bed S&S to specify acquisitions performed in a single stationary bed position. Specifically, we assessed in two phantom studies the impact of CBM on image quality, noise, axial sensitivity profiles, count statistics and quantification accuracy across a range of acquisition parameters, including scan duration, bed speed, axial position, and sensitivity mode. In the clinical part of the study, sequential CBM and single-bed S&S scans with matching scan range were performed in ten oncological patients to assess differences in axial count profiles, image noise, as well as lesion and organ quantification to determine whether the scan mode affects diagnostic image quality or clinical interpretation.

## Materials and methods

### Experimental setup and acquisition for phantom studies with tube and IEC Phantom

**Fig. 1:**
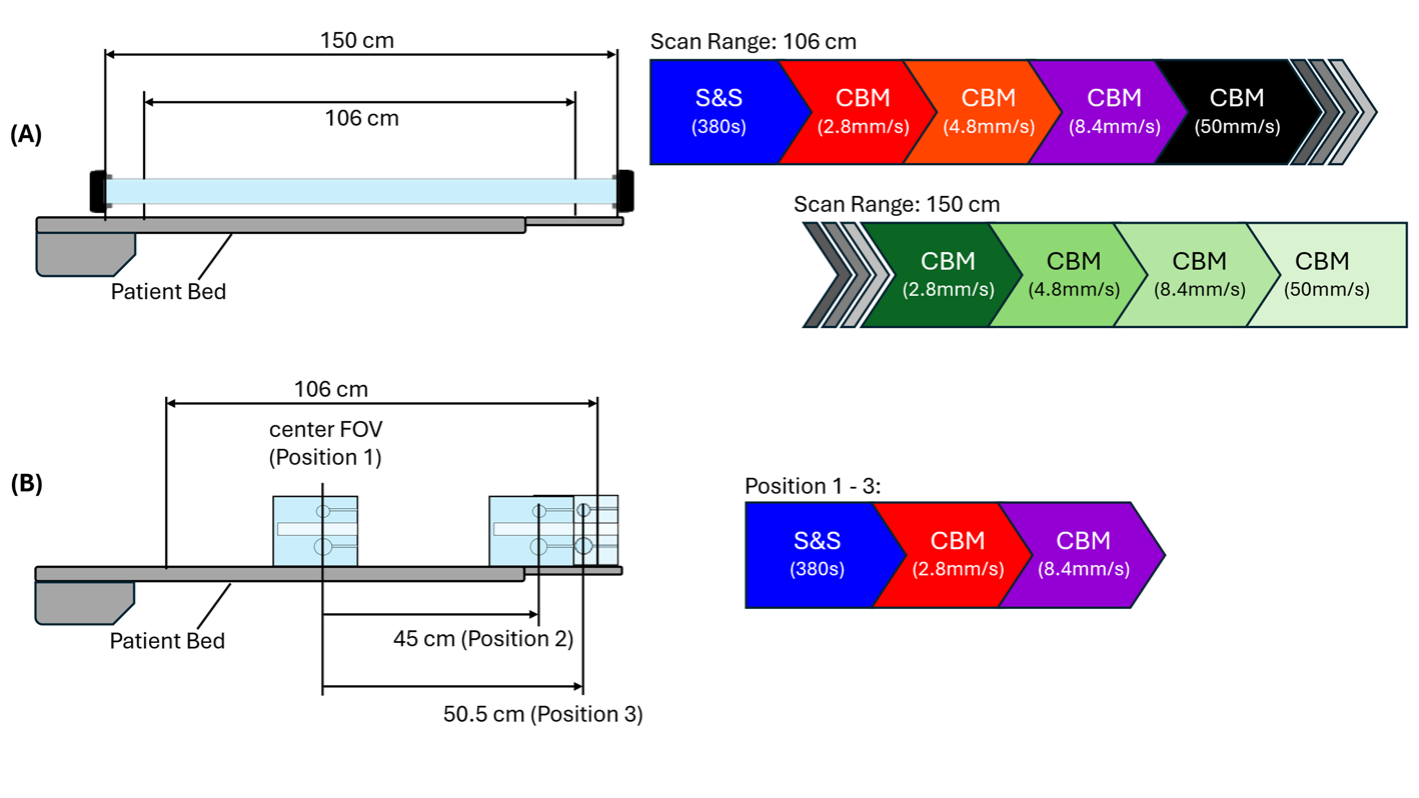
Experimental setup and scan sequence for single-bed S&S and CBM scans for **A** a 150 cm long tube phantom with 106 cm and 150 cm scan range, and **B** a NEMA IEC phantom at three different axial bed positions with 106 cm scan range

A custom-made polyvinylchloride cylindrical “tube” phantom (inner diameter: 5.5 cm; length: 150 cm) was filled with aqueous [¹⁸F]-fluoride solution (2.2 kBq/mL at scan start) and positioned centrally in the transaxial FOV (Fig. 1A). A scan in S&S mode (380 s total acquisition time) was followed by CBM scans using bed speeds of 2.8 mm/s (378 s), 4.8 mm/s (219 s), 8.4 mm/s (126 s), and 50 mm/s (21 s), each with a scan range of 106 cm, to allow direct comparison between S&S and CBM.

Subsequently, CBM scans with an extended scan range of 150 cm covering the tube phantom were acquired at the same bed speeds, resulting in respective acquisition times of 533, 310, 177, and 28 s (Fig. 1A). Here, a direct comparison with the S&S mode is not feasible for scan ranges exceeding 106 cm, as the system does not support multi-bed S&S acquisitions. Extended-range CBM acquisitions were included to characterize system behaviour for scan ranges beyond 106 cm enabled by the CBM mode.

The activity concentrations at the start of each scan and the time delays between sequential acquisitions are reported in Supplementary Table 1.

To evaluate position-dependent effects and simulate realistic tumour-to-background conditions, the NEMA IEC body phantom was used for image quality, quantification and noise assessment. The background compartment of the phantom was filled with 2.1 kBq/mL [¹⁸F]-fluoride solution and the sphere inserts were filled with 8.5 kBq/mL, resulting in a sphere-to-background ratio of 4:1. Analogous to the tube phantom study, an initial 380 s single-bed S&S acquisition was followed by CBM scans at bed speeds of 2.8 and 8.4 mm/s. All scans were sequentially performed at axial positions of 0 cm (centre), 45 cm, and 50.5 cm offset (Fig. 1B, Supplementary Table 1). For both phantom studies, CT scans (120 kVp, 50 mAs) for attenuation correction covering the respective PET scan range were performed.

An inherent limitation of the vendor implementation of CBM is that the CT acquisition range is fixed to match the PET image range (here 106 cm and 150 cm). As no attenuation information is available for lines of response (LORs) extending beyond this range, such events are excluded from image reconstruction, effectively reducing the number of usable events for quantitative CBM image reconstruction.

### Patient scans

This study includes data from a clinically heterogenous group of 10 oncological patients (3 females, 7 males; mean age: 71 ± 11 years, mean weight: 82.0 ± 14.1 kg) that received either a [^18^F]FDG or a [^18^F]PSMA-1007 clinically indicated PET/CT scan (Table 1). The patients were administered intravenously 2 MBq/kg body weight of [^18^F]FDG (mean activity: 149.7 ± 25.8 MBq; uptake time: 59 ± 4 min) or [^18^F]PSMA-1007 (mean activity: 179.0 ± 28.3 MBq; uptake time: 90 ± 3 min). Each patient underwent a diagnostic contrast enhanced (Ultravist 370, 120 mL, 2.2 mL/s) CT scan with a range of 106 cm, followed by a standard 300 s single-bed S&S scan and a CBM scan with a bed speed of 2.8 mm/s (duration: 378 s). The CBM scan range was 106 cm, equal to the single-bed S&S scan. In eight patients, the CBM scan preceded the S&S scan, while in two patients the S&S scan was performed first. The CBM speed of 2.8 mm/s was selected based on vendor recommendations to ensure image quality comparable to the standard 300 s min S&S protocol.

### Image reconstruction

All phantom and patient datasets were reconstructed using the vendor-based reconstructions (e7 tools, Siemens Healthineers, Knoxville, TN, USA). A reconstruction protocol routinely used at our institute for [^18^F]FDG and [^18^F]PSMA-1007 examinations was used, employing an Ordinary-Poisson Ordered-Subsets Expectation-Maximization algorithm with four iterations and five subsets, including point-spread-function modelling and time-of-flight information.

Standard decay correction within each acquisition was applied to account for intra-scan radioactive decay. However, decay correction between successive phantom scans could not be implemented via reframing of the CBM list-mode data. Consequently, the differences in activity concentration between phantom measurements were inherent to the sequential acquisition (Supplementary Table 1). Reconstructions were performed in both HS and UHS mode using a 440 × 440 matrix with isotropic voxel size of 1.65 mm and without post-filtering. For both phantoms, listmode data from the 380 s S&S scan were additionally reframed to 300 s to match the standard clinical S&S acquisition duration.

### Tube phantom analysis: image uniformity, noise, count statistics

The tube phantom dataset was used to evaluate the impact of S&S versus CBM on image uniformity, noise, and count statistics. Different bed motion speeds across both sensitivity modes were investigated. Image analysis was performed in MATLAB v2024b (MathWorks Inc., Natick, MA, USA).

#### Image uniformity and noise

A circular ROI (30 mm diameter) was placed in each transaxial image slice across the full axial extent of either 106–150 cm. The mean ROI activity concentration per slice was used to generate axial profiles and assess uniformity, quantified as the standard deviation of mean activity concentrations across all slices. A decay correction factor was applied on the measured mean activity to account for reduced activity concentrations at later acquisition time points (Supplementary Table 1). Similarly, axial image noise profiles were derived from these slice-wise ROIs as the coefficient of variation (CV) in percentage, defined as the ratio of voxel-wise standard deviation to mean activity concentration. To provide a single noise metric for comparison, the average CV was calculated over the central 80 cm and 124 cm for scan ranges of 106 cm and 150 cm, respectively. These restricted axial ranges were chosen to exclude the outermost image slices, which are strongly affected by increased noise due to reduced sampling. Rendering quantitative metrics derived from these regions in corresponding patients scans is less reliable because of the limited number of contributing LORs. In addition, this is motivated by the outermost ± 13 cm on either side of the FOV lacking differences due to acceptance angle between the HS and UHS mode [35].

Based on Poisson statistics, image noise is expected to scale inversely with the square root of the acquired counts, and thus directly with the square root of the bed speed (Eq. 3). To confirm this correlation is true across different sensitivity modes, bed speeds, and scan ranges, we analysed the fit of the average CV to the corresponding bed speed for both sensitivity modes and scan lengths of 106 cm and 150 cm.

#### Count statistics

Single-slice rebinning (SSRB) was performed, in which oblique LORs were projected onto the nearest transaxial slice, compressing the 3D dataset into slice-wise count distributions and thereby approximating the axial count profile [36]. Net true count rates and axial profiles were compared between S&S (300 s and 380 s) and CBM (2.8 mm/s, 106 cm and 150 cm) acquisitions. Radioactive decay between successive scans was explicitly accounted for in this analysis by applying a decay correction factor compensating for reduced activity at later acquisition times (Supplementary Table 1).

**Fig. 2:**
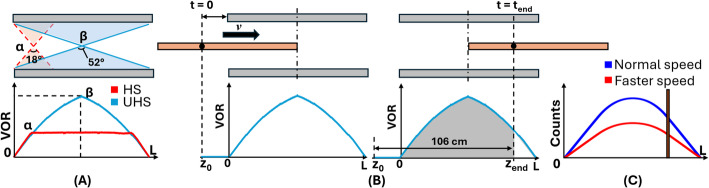
**A** Volume of response (VOR) profiles in ultra-high sensitivity (UHS, 52° axial acceptance angle) and high sensitivity (HS, 18°) modes, illustrating the difference in axial coverage and profile shape; **B** CBM acquisition showing the bed trajectory from scan start to end and how a given slice traverses the scanner’s axial FOV **C** Axial count profiles for different CBM bed speeds illustrating the smoothing effect and count reduction with increasing speed

In order to facilitate a more profound comprehension of the axial count profiles and the effects of different sensitivity modes, bed motion speeds, and scan ranges on CBM performance, in comparison with that of single-bed S&S acquisition, the theoretical basis is initially introduced: The slice sensitivity profile is proportional to the volume of response (VOR) and determined by the scanner geometry [37]. The VOR corresponds to the total number of LORs contributing to a given axial slice at a specific position, with recorded event counts being proportional to the VOR. In the Biograph Vision Quadra scanner, the sensitivity profile approximates a convex triangular shape in UHS mode and a trapezoidal shape in HS mode [18], as illustrated in Fig. 2A.

For single-bed S&S acquisitions, the number of counts *C*_*i*_ in slice *i* for a scan time $\:t$_end_ can be described by:

1 $$ C_{i} \propto \int_{0}^{{t_{{end}} }} {VOR} \left( t \right)dt $$

and simplifies under the assumption of negligible decay during acquisition (i.e. isotope half-life > > t_end_)., to:

2 $${C_i} \propto VOR \cdot {t_{end}}$$

In CBM acquisitions, *C*_*i*_ depends not only on scan duration but also on the bed speed and the trajectory of each slice through the scanner. Illustrated in Fig. 2B a slice of thickness *d*, initially located at axial position *z*_*0*_, moves through the scanner at a constant speed *v.* The bed translates the slice over the whole scan range during the acquisition time *t*_*end*_. Therefore, the count contribution for a slice is proportional to the convolution of its trajectory with the VOR according to:

3 $$ C_{i} \propto \int_{{z_{0} }}^{{z_{{end}} }} {VOR} \left( z \right) \cdot \frac{d}{v}dz $$

Here, $\:\frac{d}{v}$ represents the effective residence time of the slice at position $\:z$. As the bed speed increases, the residence time decreases, leading to lower counts per slice. Consequently, the resulting axial count profile becomes a smoothed representation of the underlying VOR and scales with the CBM table speed (Fig. 2C).

Of note, CBM employs the concept of overscan, in which additional leading and trailing segments of the object’s axial image range are translated through the scanner’s axial FOV during the scan (see Supplementary Fig. 1). Overscan is defined as a percentage of the scanner’s axial FOV and is fixed at 50% for the Biograph Vision Quadra. This setting was previously shown to provide an optimal balance between acquisition time and image quality, with 50% overscan closely matching the sensitivity profiles of CBM and overlapping S&S scans for SAFOV PET scanners [30]. The extended scan range (image range plus the overscan range) is applied only to the PET acquisition, while the CT acquisition remains limited to the PET image range (see discussion section for details).

### IEC phantom analysis: image quality and contrast recovery

The NEMA IEC body phantom data were analysed to evaluate potential position and speed dependent image degradation for both sensitivity modes due to the CBM protocol. Images were visually compared between S&S (300 s), CBM at 2.8 mm/s, and CBM at 8.4 mm/s for positions at the scanner centre and axial offsets of 45 cm and 50.5 cm. Image noise was quantified analogously to the tube phantom using the CV, calculated from a box shaped volume of interest (VOI) (150 × 25 × 135 mm^3^) in the background compartment, delineated and analysed using AMIDE software version 1.04 [38]. To assess whether CBM acquisitions introduce challenges for scatter or attenuation correction, residual activity in the lung insert was evaluated. For this, a cylindrical VOI (30 mm diameter, 135 mm length) was placed within the lung insert, and the lung residual error as the ratio of its mean activity concentration to the mean of the background VOI was determined. The image quality was further assessed by means of contrast recovery. Spherical VOIs corresponding to the phantom sphere diameters (10, 13, 17, 22, 28, and 37 mm), were placed on the respective spheres. Contrast recovery coefficients (CRCs) were calculated as the measured sphere-to-background (SBR) activity concentration ratio, normalized to the SBR of 4.

### Analysis of patient images

#### Count statistics and image noise

SSRB was applied to the sinograms of all datasets to extract both slice-wise and total net true count statistics. To identify potential differences in count statistics or distribution across the axial FOV axial net true count profiles and total net true counts were compared between single-bed S&S and CBM acquisitions. To assess image noise, a spherical VOI with a 30 mm diameter was placed within the liver to derive the CV in both CBM and S&S datasets, as a metric for image noise in a reference area with homogenous physiological uptake.

#### Lesion quantification and clinical scoring

Lesions were independently delineated on the S&S and CBM datasets by an experienced nuclear medicine physician using a 50% SUV_max_ isocontour threshold in a clinical imaging reviewing software (Affinity 3.0.5, HQ Hermes Medical Solutions AB, Stockholm, Sweden). For each lesion and liver reference VOI, SUV_mean_ and SUV_max_ values from S&S and CBM were compared and statistically analysed. In addition, for the patients that underwent a [^18^F]PSMA-1007 scan, PSMA expression scores analogue to the PROMISE criteria [39] categorizing a lesion’s uptake according to the PSMA uptake in the blood pool, the spleen and the salivary glands were determined. Resulting scores were compared across scan modes to assess the potential clinical impact of differences in SUV values.

#### Statistical analysis

Normality of the variables describing differences between CBM and S&S scans was evaluated using the Shapiro–Wilk test [40] (α = 0.05), respectively. For variables meeting the assumption of normality, paired *t*-tests were performed to assess statistically significant differences between the two modes (α = 0.05). The size effect at paired *t*-test was quantified with Cohen’s d [41] and for small sample correction (*n* < 20) Hedges’ g [42] was applied. In cases where the normality assumption was not satisfied, the Wilcoxon signed-rank test was used as an alternative for assessment of statistical significance. All statistical analyses and graphical representations were performed using SPSS Statistics, version 29.0 (IBM Crop., Armonk, NY, USA) and MATLAB v2024b (MathWorks Inc., Natick, MA, USA).

## Results

### Tube phantom: image uniformity, noise and count statistics

#### Image uniformity

**Fig. 3:**
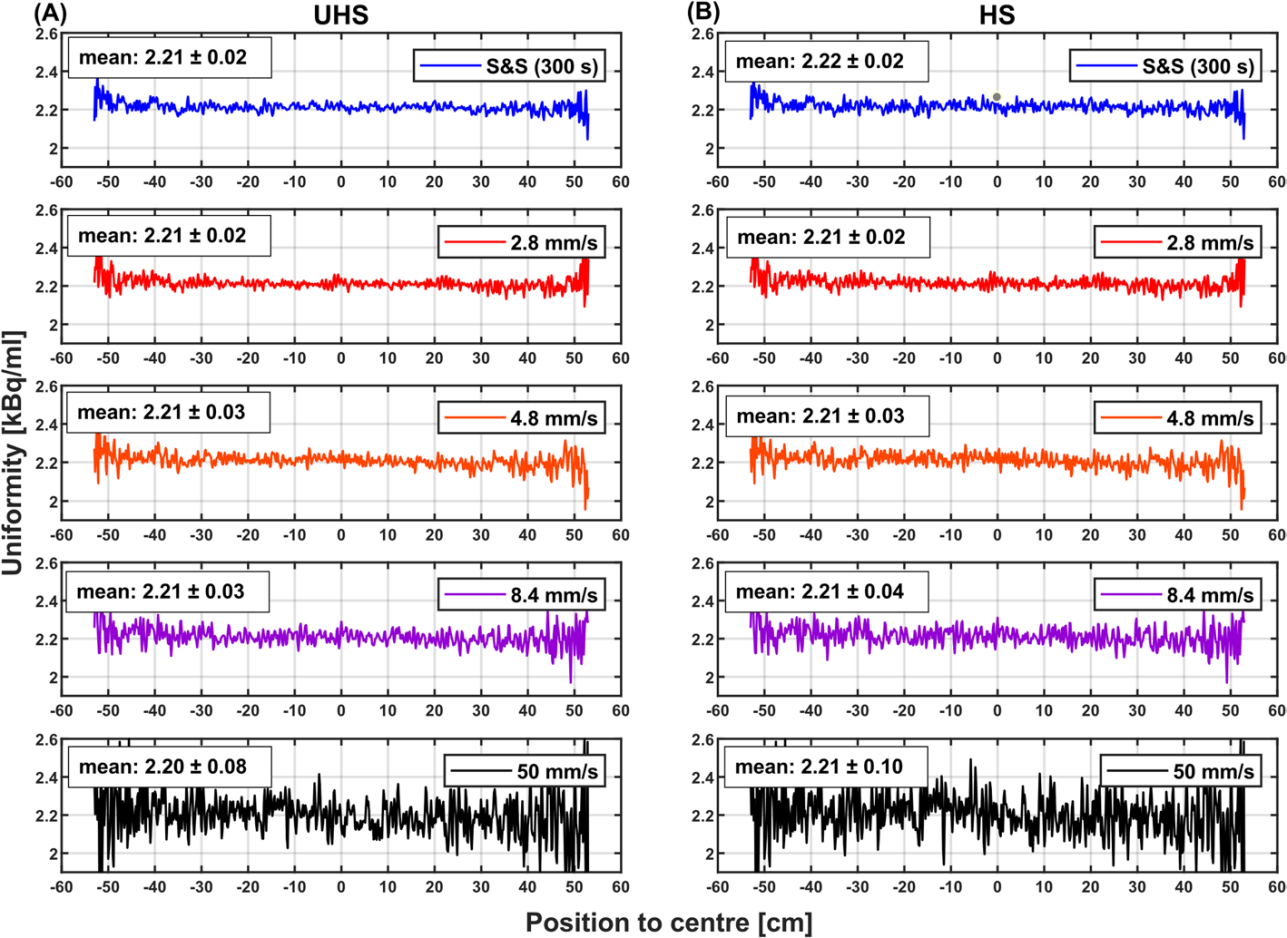
Axial activity concentration profiles from the tube phantom for S&S (300 s) and CBM (106 cm scan range) at different bed speeds for **A** UHS and **B** HS mode. Mean values and standard deviations are indicated

A comparable uniformity was obtained for S&S and the 2.8 mm/s CBM acquisition with mean activity concentrations of 2.21 ± 0.02 and 2.21 ± 0.02 kBq/mL, respectively (UHS mode, Fig. 3A). The uniformity decreased towards higher bed speeds, yielding 2.21 ± 0.03 (4.8 mm/s), 2.21 ± 0.03 (8.4 mm/s), and 2.20 ± 0.08 kBq/mL (50 mm/s), however, the variation remained acceptably low. A similar trend was observed in HS mode (Fig. 3B).

#### Image noise

**Fig. 4:**
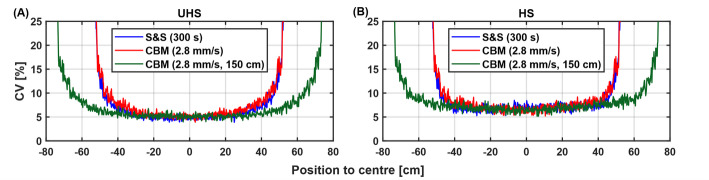
Axial CV (%) noise profiles for tube phantom with S&S (300 s) and CBM (2.8 mm/s) at scan ranges of 106 cm and 150 cm for **A** UHS and **B** HS mode

The axial noise profiles in Fig. 4 show no notable differences between CBM (2.8 mm/s, 106 cm, 378 s) and S&S (300 s) acquisitions and demonstrate an extended central low-noise region within the 150 cm CBM scan. In UHS mode, average CV values across the central 80 cm were comparable for S&S with 300 s (5.4%) and CBM with 378 s (5.9%), and moderately higher than for S&S with 380 s (4.8%). In HS mode, the discrepancy between S&S and CBM was slightly less pronounced, with CV values of 6.9% and 7.1% for S&S (300 s) and CBM (378s), respectively (Fig. 4).

**Fig. 5:**
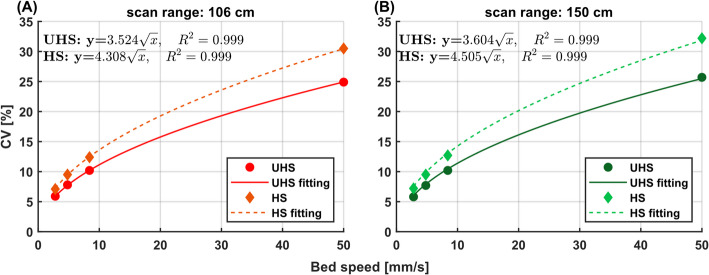
Correlation between average coefficient of variation (CV, %) for central axial regions in CBM acquisitions with bed speeds from 2.8 mm/s to 50 mm/s for scan ranges of **A** 106 cm and **B** 150 cm

An increase in bed speed resulted in higher average CV, with HS mode consistently exhibiting higher CV values than UHS. Similar trends across sensitivity modes and bed speeds remained evident with extended scan range of 150 cm. With scan range extension, average CVs were comparable to those observed at the 106 cm scan range with 7.1%, 9.5% 12.4% and 30.5% (HS, 106 cm) and 7.2%, 9.5%, 12.7% and 32.2% (HS, 150 cm). The relationship between bed speed and image noise was well described by the square root function (Fig. 5) with a fit accuracy of *R* = 0.999 for both sensitivity modes and scan ranges.

#### Net true counts and axial profiles

**Fig. 6:**
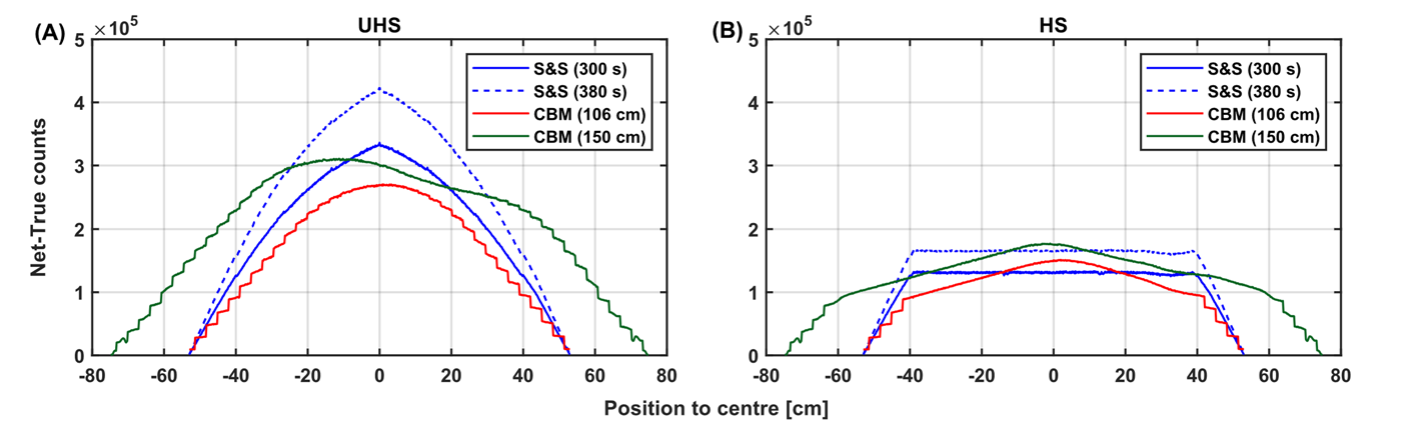
Axial true count profiles of the tube phantom acquired with S&S (300 s and 380 s) and CBM with 106 cm and 150 cm scan ranges for UHS (**A**) and HS mode (**B**)

Net true counts aligned with the observed noise and uniformity trends. When comparing S&S (300 s) to CBM (2.8 mm/s, 106 cm, 378 s), total net trues are decreased by 17.7% in UHS mode (from 131.9 × 10^6^ to 108.6 × 10^6^ and 7.5% in HS mode (from 73.9 × 10^6^ to 68.3 × 10^6^). Figure 6 presents the axial true count profiles (inter-scan decay corrected) in both modes. In UHS mode (Fig. 6A), a distinct reduction in the peak of axial counts is observed for CBM (2.8 mm/s, 378 s) compared to S&S (380 s), with a central count decrease of 36.7% (from 4.2 × 10⁵ to 2.7 × 10⁵). In contrast, HS mode (Fig. 6B) shows smaller reductions. Notably, in HS mode, the CBM acquisition disrupts the flat plateau typically seen across the central 80 cm—indicative of a homogeneous sensitivity profile. Instead, CBM profiles show rounded peaks in UHS mode, slightly elevated central regions in HS mode, and introduce a stair-step pattern. The 150 cm CBM scan in UHS mode also reveals an asymmetric distribution (Fig. 6A).

### IEC phantom: image quality, noise and contrast recovery

**Fig. 7:**
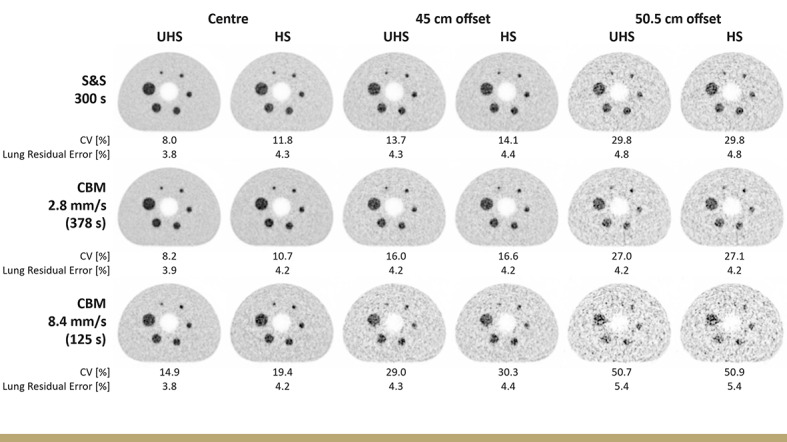
Transaxial image slices of the IEC phantom acquired with S&S and CBM protocols at 2.8 and 8.4 mm/s, shown at different axial positions and in both sensitivity modes. Image noise is annotated as coefficient of variation (CV, %)

Figure 7 demonstrates that the 2.8 mm/s CBM protocol (378 s) preserved comparable image quality to the S&S protocol (300 s) across all axial positions and for both sensitivity modes. This was supported by similar image noise levels, e.g., in UHS mode, CV values at the scanner centre were 8.0% (S&S) and 8.2% (CBM), and at the 45 cm offset position 13.7% and 16.0%, respectively. Increasing the bed speed to 8.4 mm/s substantially elevated image noise to 14.9% (centre) and 29.0% (45 cm) and impaired detectability of the smallest (10 mm) sphere at the 50.5 cm offset position.

**Fig. 8:**
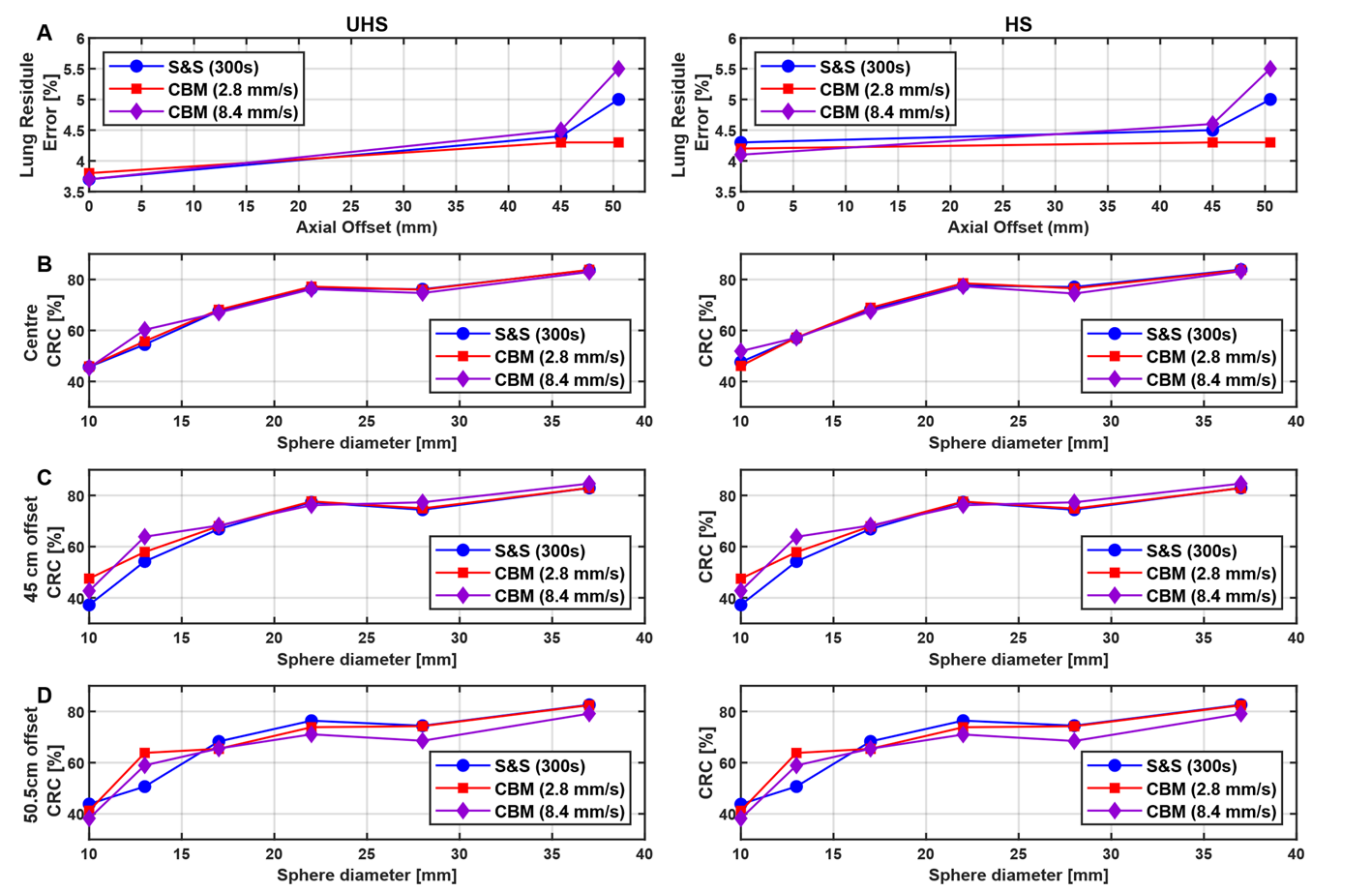
**A** IEC phantom lung residual error for S&S and CBM with 2.8 and 8.4 mm/s speeds at different axial positions and UHS and HS sensitivity modes; **B–D** Analog comparison for contrast recovery coefficients (CRC) for the six spheres

The lung residual error (Fig. 8A) was slightly higher in HS mode than in UHS at the axial centre (4.3% vs. 3.8%) and increased modestly toward the edges (up to 5.4% at 50.5 cm). 2.8 mm/s CBM scans matched the S&S reference across all positions, indicating no adverse impact on scatter or attenuation correction. A minor increase in lung residual error was observed only under the lowest count condition, i.e., at the 50.5 cm offset position with a fast CBM speed of 8.4 mm/s (5.4% vs. 4.2% at 2.8 mm/s). As demonstrated in Fig. 8B-D, CRC values across all sphere sizes and sensitivity modes were comparable between S&S and CBM protocols at both the centre and the 45 cm offset positions. Only at the outermost position (50.5 cm) a modest decline in CRC was observed for the 8.4 mm/s CBM, particularly for medium and large spheres. For example, in UHS mode, the CRC for the 37 mm sphere decreased from 83% (S&S) to 79% (8.4 mm/s CBM), and for the 22 mm sphere from 76% (S&S) to 71%, respectively.

### Patients study

#### Image quality

**Fig. 9:**
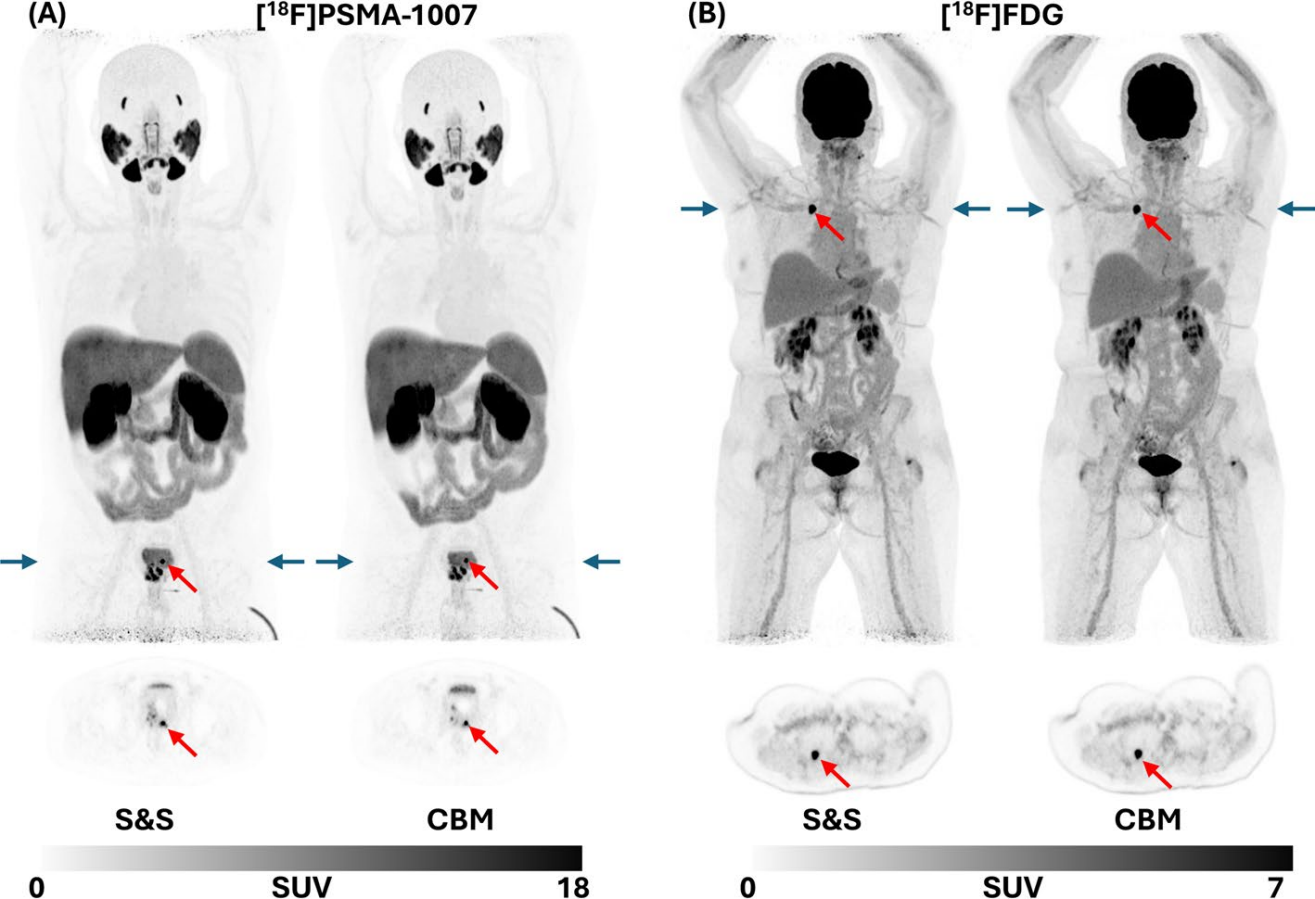
Maximum intensity projections and transaxial PET images of representative patients (**A** patient 1, 226 MBq [^18^F]PSMA-1007 with prostate cancer in a primary staging setting ; **B** patient 9, 174 MBq [^18^F]FDG with lung cancer in a primary staging setting) acquired with S&S (300 s) and CBM (2.8 mm/s, 378 s) in UHS mode. Exemplary suspicious malignant lesions (red arrows) show visually comparable appearance between S&S and CBM PET images

A representative [^18^F]PSMA-1007 and a [^18^F]FDG case are shown in Fig. 9 for visual comparison of the sequential S&S and CBM scans. Comparable image quality was obtained, as reflected by similar CV values 4.7% (S&S) vs. 5.8% (CBM) for the [^18^F]PSMA-1007 case, and 8.6% for both modes in the [^18^F]FDG case. CBM showed marginally lower noise at the edges of the image range. Lesion conspicuity and quantification were preserved for these two patients across scan modes, with similar SUV_mean_ values: 39.6 (S&S) vs. 37.1 (CBM) for [^18^F]PSMA-1007 and 12.0 (S&S) vs. 12.1 (CBM) for [^18^F]FDG. These findings were consistent across all patients, with no observed loss in diagnostic image quality or lesion detectability when using CBM acquisitions.

#### Image noise and counts statistics

**Fig. 10:**
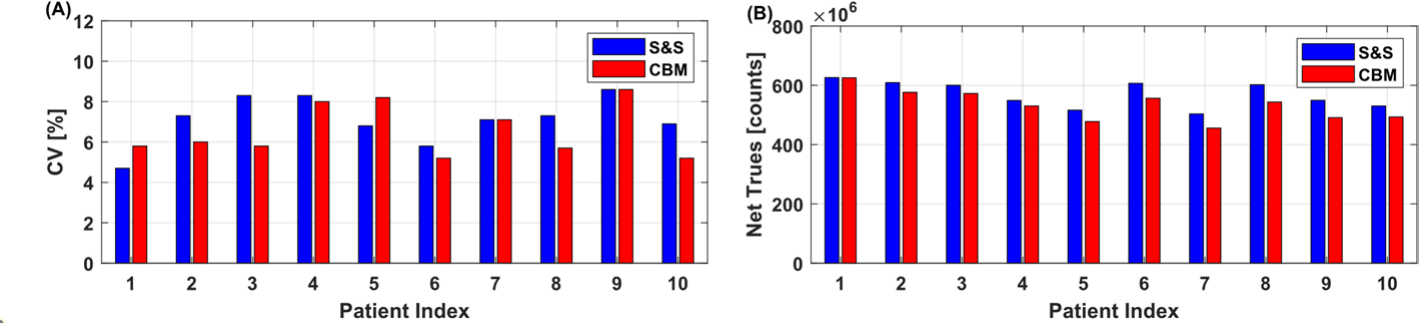
Comparison of coefficient of variation (CV, %) (**A**) and net trues counts (**B**) for single-bed S&S and CBM acquisitions in ten oncological patients with values listed in Table 1

Across all patients, image noise between both modes was comparable with mean CV values of 6.6 ± 1.3% (CBM) and 7.1 ± 1.2% (S&S) (see Fig. 10A for patient individual CV comparison). The difference in CV was normally distributed (Shapiro–Wilk test, *p* = 0.87) and not statistically significant (paired *t*-test, *p* = 0.20)). Total net true counts for the 380 s CBM acquisition (532.2 ± 50.0 × 10^6^) were slightly lower, showing a mean deviation of − 6.6 ± 3.1% compared to the 300 s S&S scan (569.3 ± 42.0 × 10^6^) (see Fig. 10B for patient net true counts comparison). The difference was normally distributed (*p* = 0.59), and a paired *t*-test confirmed statistical significance (*p* < 0.01).

**Fig. 11:**
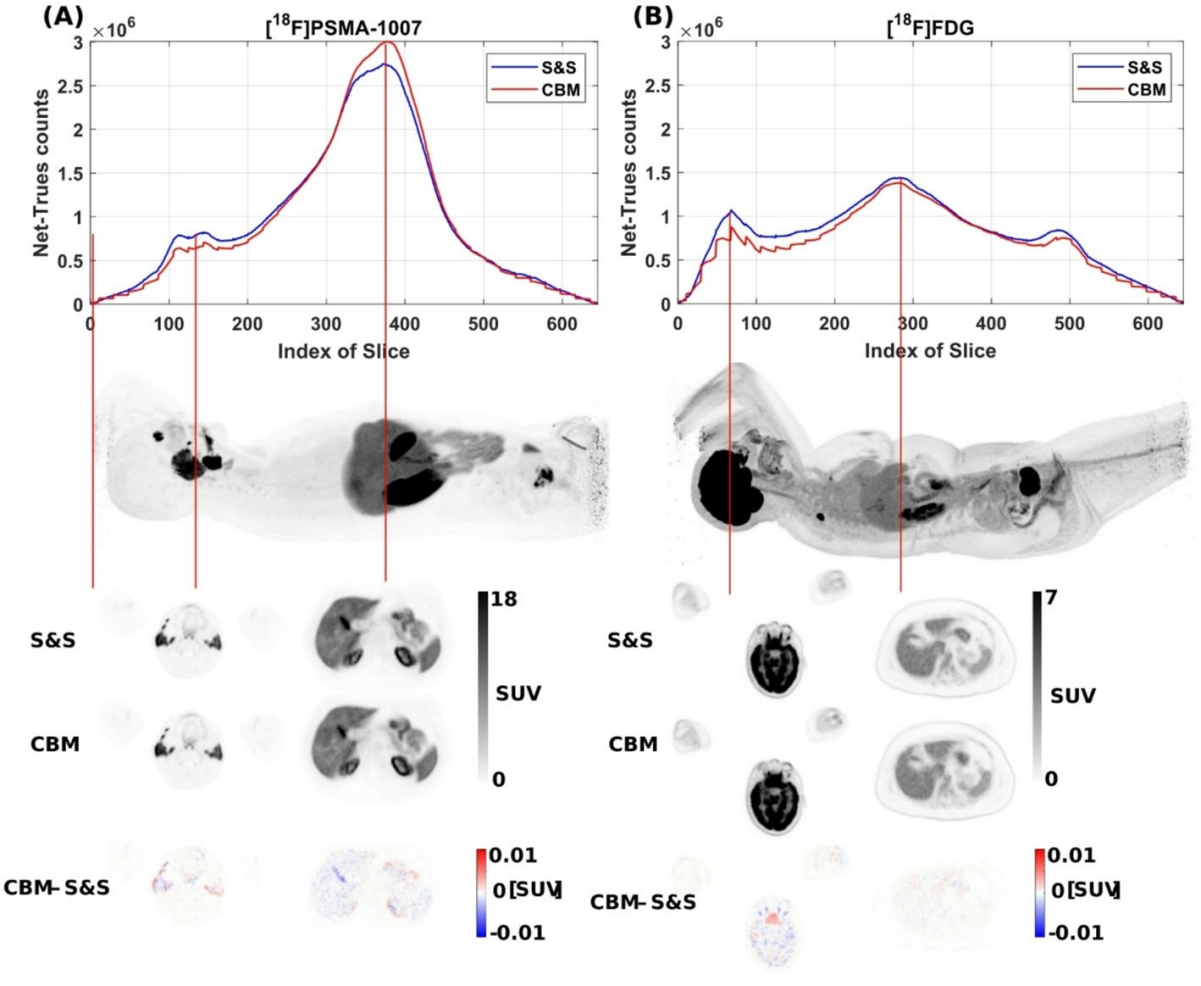
Net-trues slice profile and sagittal MIP view of representative patients (**A** patient 1, 226 MBq [^18^F]PSMA with prostate cancer; **B** patient 9, 174 MBq [^18^F]FDG with lung cancer). Total true counts were 6.26 × 108 (S&S) vs. 6.25 × 108 (CBM) at patient 1, and 5.50 × 10^8^ (S&S) vs. 4.91 × 10^8^ (CBM) for patient 9, acquired with S&S (300 s) and CBM (2.8 mm/s, 378 s) in UHS mode. Transaxial images and absolute difference images (CBM - S&S) of peak uptake regions —salivary glands and upper abdomen for [¹⁸F]PSMA-1007, and brain and upper abdomen for [¹⁸F]FDG— exhibit no relevant differences between S&S and CBM

Agreement between CBM and S&S acquisitions extended beyond the global metrics total counts and image noise to the axial distribution of net true counts, as demonstrated in two representative cases shown in Fig. 11. These examples are particularly illustrative due to their distinct tracer uptake patterns: the [^18^F]PSMA-1007 case exhibited high uptake in the centre FOV (upper abdomen and pelvic region) as well as in the salivary glands, while the [^18^F]FDG case displays the physiologically high uptake in the brain, located at the distal edge of the axial FOV. In both cases, axial count profiles closely matched in shape and magnitude across both scan modes. Even at peak uptake regions, absolute slice-wise counts remained comparable, e.g., 3.0 × 10^6^ counts (S&S) vs. 2.7 × 10^6^ counts (CBM) in the abdominal region of the [^18^F]PSMA-1007 case, and 1.1 × 10^6^ counts (S&S) vs. 0.9 × 10^6^ counts (CBM) in the brain region of the [^18^F]FDG case. The difference images of the high uptake regions demonstrate negligible differences in SUV between CBM and S&S scans. An overview of individual patient CV values and net true count rates is provided in Table 1.

**Table 1:** Characteristics, scan sequence and metrics for single-bed S&S and CBM acquisitions in ten oncological patients

Patient index	1	2	3	4	5	6	7	8	9	10
Tracer	PSMA-1007	PSMA-1007	PSMA-1007	FDG	PSMA-1007	PSMA-1007	FDG	PSMA-1007	FDG	PSMA-1007
Gender	Male	Male	Male	Female	Male	Male	Female	Male	Female	Male
Weight [kg]	108	101	79	58	76.2	79.4	79.7	89	83.2	66
Age	56	68	78	87	80	64	65	78	56	82
Activity [MBq]	226	206	170	114	160	177	161	182	174	132
Scan sequence	CBM S&S	S&S CBM	CBM S&S	CBM S&S	CBM S&S	CBM S&S	CBM S&S	S&S CBM	CBM S&S	CBM S&S
CV [%] (S&S)	4.7	7.3	8.3	8.3	6.8	5.8	7.1	7.3	8.6	6.9
CV [%] (CBM)	5.8	6.0	5.8	8.0	8.2	5.2	7.1	5.7	8.6	5.2
Net Trues (S&S)	626.15 M	609.17 M	600.16 M	549.15 M	516.38 M	606.70 M	503.60 M	602.04 M	549.51 M	530.39 M
Net Trues (CBM)	625.60 M	576.32 M	572.47 M	530.46 M	477.77 M	556.30 M	455.86 M	543.64 M	490.98 M	493.42 M
PSMA Score (S&S)	2	1	1	-	3	1	-	1	-	1
PSMA score (CBM)	2	1	1	-	3	1	-	1	-	1

Quantitative metrics include coefficient of variation (CV, %) in the liver, total net true counts (in millions), and PSMA expression scores for representative lesions in [¹⁸F]PSMA-1007 patients)

#### SUV and clinical scoring

**Fig. 12:**
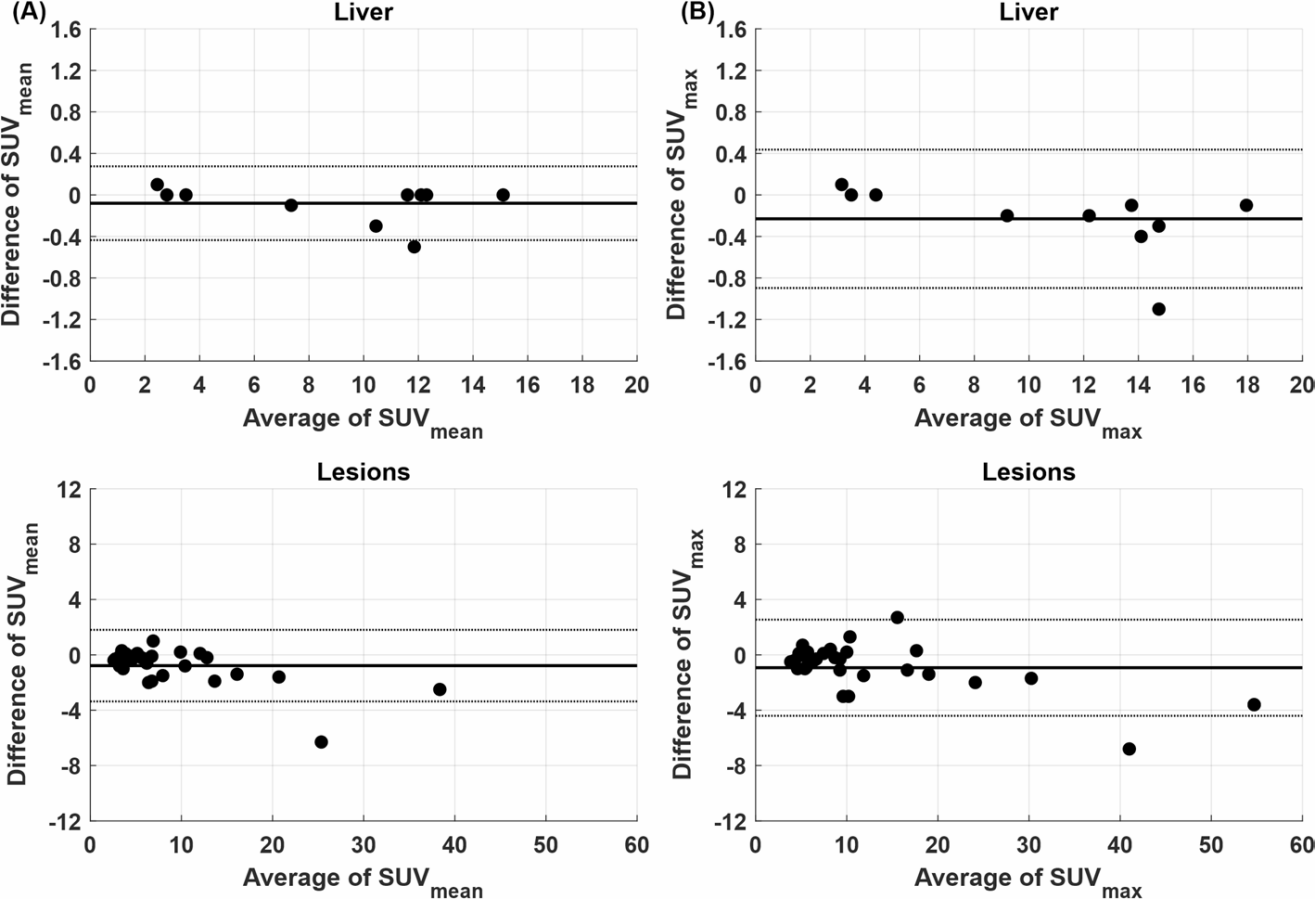
Bland–Altman plots showing differences of SUVmean (**A**) and SUVmax (**B**) between S&S and CBM scans relative to their common mean value for both liver and lesion

Comparable SUV metrics were obtained in the liver for both acquisition modes. Mean SUV_mean_ values were 11.6 ± 2.1 (S&S) | 11.5 ± 2.2 (CBM) and 2.9 ± 0.5 (S&S) | 2.9 ± 0.4 (CBM) for the [^18^F]PSMA-1007 and [^18^F]FDG scans, respectively. The average deviation (together for both tracer examinations) between S&S and CBM was − 0.4 ± 2.1% (*p* = 0.20) and − 1.4 ± 2.5% (*p* = 0.02) in SUV_mean_ and SUV_max_, respectively. Bland–Altman analysis (Fig. 12A/B) supported this agreement, revealing minimal bias between scan modes with mean differences between CBM and S&S of − 0.08 ± 0.18 (SUV_mean_) and − 0.23 ± 0.34 (SUV_max_).

Across 30 delineated lesions, SUV values were slightly lower in CBM compared to S&S acquisitions. Mean SUV_mean_ values were 9.4 ± 8.4 (S&S) | 8.5 ± 7.5 (CBM) and 6.4 ± 4.0 (S&S) | 6.2 ± 4.2 (CBM) for the [¹⁸F]PSMA-1007 and [¹⁸F]FDG scans, respectively. The average deviation (together for both tracer examinations) between S&S and CBM was − 7.4 ± 9.9% (*p* < 0.001) and − 5.2 ± 10.6% (*p* = 0.004) in SUV_mean_ and SUV_max_, respectively. Bland–Altman analysis (Fig. 12) confirmed this tendency, showing more pronounced differences than in the liver, with mean reductions of − 0.8 ± 1.3 for SUV_mean_ and − 0.9 ± 1.8 for SUV_max_ in CBM relative to S&S. These differences, however, remained within a range that is not considered to have impact on clinical interpretation or diagnostic decision-making, which is supported by the consistent PSMA expression scores (Table 1) for both S&S and CBM acquisitions.

## Discussion

This study systematically characterized the impact of CBM on image quality and quantitative performance in the Biograph Vision Quadra LAFOV PET/CT scanner with both phantoms and patient data. The image quality metrics and the trade-offs by different acquisition parameters between CBM and single-bed S&S were quantified.

Phantom experiments confirmed that the high axial uniformity of in-plane activity concentration of the S&S scan (2.21 ± 0.02 kBq/mL) was maintained in CBM acquisitions up to 50 mm/s bed speed. A slight increase in axial variation was observed with higher bed speeds, e.g., 2.21 ± 0.02 kBq/mL (2.8 mm/s) vs. 2.20 ± 0.08 kBq/mL (50 mm/s), which can be attributed to the reduced counts from shorter acquisition times. This was supported by increasing image noise with bed speed, e.g. in UHS mode with a CV of 5.9% (2.8 mm/s) to 10.2% (8.4 mm/s) and 8.2% (2.8 mm/s) to 14.9% (8.4 mm/s) for the tube and IEC phantom, respectively. The image noise averaged over the central 80 cm aFOV was reduced for the tube phantom (Fig. 4) by a factor of 1.28 (CV: 6.9% (HS), 5.4% (UHS)) in S&S and 1.20 (CV: 7.1% (HS), 5.9% (UHS)) in CBM. For S&S, this improvement in image noise due to higher sensitivity has been reported previously [35] and is object-dependent, as oblique LORs are more likely to be attenuated when γ-rays traverse longer distances through the patient or phantom. In CBM, the stronger weighting toward direct, higher-quality data planes results in a larger difference compared to S&S in UHS mode with more oblique data being captured compared to HS mode. Similar to S&S, CBM yields a flatter axial sensitivity profile in HS mode (Fig. 6A), with the selection of sensitivity mode therefore requiring a trade-off between maximum sensitivity (UHS) and a more homogeneous sensitivity distribution (HS) along the axial FOV.

Notably, independent of the scan range, bed speed and sensitivity modes, image noise scaled proportionally to the square root of the bed speed, enabling straightforward CV based protocol optimization analogous to scan time adjustments in conventional S&S acquisitions. This correlation between bed speed and image noise applies not only to the LAFOV Biograph Vision Quadra but also appears to hold for its SAFOV counterpart, the Biograph Vision 600, where a CV increase from ~ 6% to ~ 16% was reported as bed speed increased from 0.5 to 3.3 mm/s [43].

While image noise increased with bed speed, the IEC phantom study demonstrated that CBM does not impair quantitative accuracy in terms of contrast recovery compared to single-bed S&S even across different axial positions and varying bed speeds. CRC values across all sphere sizes and sensitivity modes were comparable at both the centre and the 45 cm offset positions. Even at the outermost axial position (50.5 cm) and the highest bed speed of 8.4 mm/s, only a minor CRC reduction was observed, e.g., from 76% (S&S) to 71% (CBM) for the 22 mm sphere, underscoring that quantitative accuracy remains robust despite the presence of increased image noise. These findings align with previous SAFOV PET/CT studies, which also reported comparable CRC values between CBM and S&S for equivalent net-true statistics (Owaki et al. [44], 10 mm sphere: 54% (S&S) vs. 53% (CBM)) or equivalent acquisition times (Yamashita et al. [45], 13 mm sphere: 43% (S&S) vs. 42% (CBM)). To obtain comparable image noise, CBM required a longer acquisition time (378 s) than S&S (300 s). Consequently, at identical acquisition times, CBM showed higher noise, with CV values of 5.9% vs. 4.8% for S&S. Similar increases have been reported for the Biograph mCT, where image noise increased from 3.3% to 3.8% [45] and from 2.2% to 2.9% [31] for S&S compared to CBM. For patient examinations, the extended 378 s CBM duration was likewise necessary to match the event statistics of the 300 s S&S scans, resulting in only a slightly lower total true count for CBM (‑6.6%). Comparable image quality and liver noise were observed, with CV values of 6.6 ± 1.3% (CBM) vs. 7.1 ± 1.2% (S&S). These findings are consistent with Schatka et al., who reported negligible liver CV differences between CBM and S&S on the Biograph mCT SAFOV PET/CT scanner (16% vs. 17%) [32].

Interestingly, in some patients, CBM scans exhibited lower liver CV than S&S despite having fewer total true counts. This reflects that liver CV represents local noise, whereas total true counts are a measure of global event statistics. For example, in patient 9 (see Fig. 9B for image noise), both CBM and S&S yielded identical CV values of 6.8% with comparable local counts at the same axial position of the liver (see Fig. 12B for axial count profiles), despite higher total true counts 550 × 10⁶ (S&S) vs. 491 × 10⁶ (CBM).

Furthermore, CBM facilitates smoother randoms estimation by leveraging axial smoothing and modelling of transverse efficiencies across image planes which can reduce image noise [30].

In theory, the prolonged CBM acquisition time in this study should have resulted in more acquired PET events compared to the S&S scan. In addition, slices at the edges of the image range should contain a higher number of events than for the S&S scan, since during the CBM scan the object passes through the highly sensitive centre FOV. This should result in a flatter axial count profile with elevated edges as illustrated in Supplementary Fig. 2A. However, CBM does not exhibit this idealized profile in practice due to the limited CT scan range, which is not extended to cover the entire CBM acquisition including the overscan range, but instead matches the PET image range. As shown in Supplementary Fig. 2B, events with LORs outside the CT scan range are excluded from reconstruction, since no attenuation correction can be applied to them [37] – which would otherwise compromise quantitative accuracy.

Theoretically, the idealized CBM profile could be achieved if no attenuating structures extend beyond the 106 cm PET axial FOV, e.g., in pediatric patients shorter than 106 cm, or in cases where LORs extend beyond the patient’s head into non-attenuating air. Even in the presence of attenuating structures beyond 106 cm, this could in principle be addressed by extending the CT acquisition to the PET scan range (approx. 1.5 times the PET image range) or by employing synthetic µ-maps for regions outside the CT coverage, e.g., derived from non-attenuation-corrected PET data [46]. In practice, however, current image reconstruction is always limited to the CT range. Therefore, substituting a standard 106 cm S&S scan with CBM to achieve a more uniform axial count profile is currently not a meaningful option and will remain so until such approaches are implemented. Nevertheless, the 106 cm CBM scan was valuable for this study being the only option to allow fair comparability to assess the impact of CBM relative to S&S.

Another deviation from the ideal profile is the staircase pattern observed at the axial edges of the CBM count profiles (Fig. 6), which has also been reported in [30] and is attributed to the data organization with a span of 19 for sinogram compression. In contrast to S&S mode, where edge slices are formed from a progressively increasing number of contributing LORs, CBM constructs edge slices from complete span 19 segments (see Supplementary Fig. 3), resulting in a stepwise sensitivity increase every 19 image slices. Of note, the asymmetric profile with a local bump ~ 15 cm off centre in the 150 cm CBM acquisition (Fig. 6A) is caused by a locally reduced bed speed near the patient bed pedestal. For long CBM scans where the total transition range would exceed the physical limitation of the bed’s transition range, the 50% overscan range cannot be fully applied. Therefore, the overscan range is reduced near the patient bed pedestal. To compensate for this shorter overscan range, the bed speed is automatically reduced, thereby preserving comparable count statistics relative to the opposing side with a full 50% overscan range.

In the patient scans with sequential CBM and single-bed S&S acquisitions minor differences in axial count profiles, total count rates and image noise did not translate into clinically relevant changes in liver or lesion SUV values for the CBM scans. The differences can be attributed to patient motion between scans, variability in delineation and inherent differences introduced by repeated acquisitions and image reconstruction, reflecting the statistical nature of PET. Bland–Altman analysis (Fig. 12) showed that SUV values were, on average, slightly lower for CBM, with low mean biases of − 0.8 and − 0.1 for lesions and liver, respectively. This trend likely reflects the scan order, as CBM preceded S&S scans in 8 of 10 patients, resulting in longer uptake times for the S&S scans. Similar findings were reported by Yamashita et al. when CBM was performed first [45], whereas Schatka et al., using randomized scan order, observed negligible differences between CBM and S&S of 0.01 and 0.03 for lesions and muscle, respectively [32]. Most SUV values remained well within the narrow limits of agreement, supporting the clinical equivalence of CBM and S&S. This is further confirmed by the unchanged PSMA expression scores across both scan modes.

As outlined previously, the 106 cm CBM acquisitions in this study served as a benchmark to enable direct comparison with standard single-bed S&S scans under matched conditions. In clinical practice, however, such a substitution is not applicable. On the Biograph Vision Quadra, overlapping S&S acquisitions are not supported. Thus, CBM is the only available option to extend the scan range beyond 106 cm. Bed speed can be adjusted for different parts of the scan range, which is typically performed to have a faster acquisition for the lower limbs (e.g., 4.8 mm/s at our institute) while using a slower speed for the head and torso (e.g., 2.8 mm/s). Our findings suggest that image uniformity and quantitative accuracy are well maintained for such multi-speed CBM protocols. In this example, using a faster 4.8 mm/s speed for the lower limbs largely reduces acquisition time by 42% with an acceptable 31% increase in image noise, (derived from square root dependence of noise on bed speed, Fig. 5).

## Conclusions

In this study, we systematically assessed the impact of continuous bed motion (CBM) on image quality, noise, and quantitative performance in the Biograph Vision Quadra LAFOV PET/CT scanner. In both phantom studies CBM preserved contrast recovery and image uniformity across all evaluated bed speeds, scan ranges, positions and sensitivity modes, showing quantitative performance comparable to conventional single-bed step‑and‑shoot (S&S) acquisitions. To obtain comparable count statistics and image noise, a prolonged acquisition was required for CBM (378 s at 2.8 mm/s bed speed) to match the S&S (300 s) protocol. Minor differences in axial count profiles, net true counts, and SUV values were observed in the patient study but did not translate into clinically relevant effects. Under the current vendor implementation, the reconstructed PET image range in CBM is constrained to the CT coverage, such that CBM does not provide an advantage over S&S for improving axial uniformity within a fixed 106 cm scan range. The findings in this study indicate that prolonged CBM acquisitions provide quantitative performance equivalent to S&S and can be applied for the scan range extension beyond the 106 cm single‑bed coverage of the Biograph Vision Quadra without compromising diagnostic image quality or quantitative accuracy.

## Supplementary Information

Supplementary Material 1.

## Data Availability

The datasets used and/or analysed during the current study are available from the corresponding author on reasonable request.
