# Supplementary material for: Continuous bed motion versus step-and-shoot acquisition in LAFOV PET/CT: insights from multi-phantom and patient studies
Source: EJNMMI Phys. 2026 Apr 12;13:33. doi: 10.1186/s40658-026-00867-3 (PMC13076829; doi:10.1186/s40658-026-00867-3)
Supplement: Supplementary file 1 — Supplementary Material 1. [file 40658_2026_867_MOESM1_ESM.docx]

# Continuous Bed Motion versus Step-and-Shoot Acquisition in LAFOV PET/CT: Insights from Multi-Phantom and Patient Studies

**Supplementary Material**

P.M. Linder^1, †^, W. Lan^1, †^, E. Calderón^1^, I. Rausch^2^, C. la Fougère^1,3^, F. P Schmidt^1,4,*^

^1^Department of Nuclear Medicine and Clinical Molecular Imaging, University hospital Tuebingen, Tuebingen, Germany

^2^QIMP Team, Center for Medical Physics and Biomedical Engineering, Medical University of Vienna, Waehringer Guertel 18-20/4L, 1090 Vienna, Austria

^3^Cluster of Excellence iFIT (EXC 2180) “Image Guided and Functionally Instructed Tumor Therapies”, University of Tuebingen, Tuebingen, Germany

^4^Werner Siemens Imaging Center, Department of Preclinical Imaging and Radiopharmacy, Eberhard Karls University Tuebingen, Roentgenweg 13, 72076 Tuebingen, Germany

^†^ Equally contributed

* Corresponding author, Email: F.Schmidt@med.uni-tuebingen.de

ORCID:

P.M. Linder: 0000-0002-0615-4137

W. Lan: 0000-0001-5453-6351

E. Calderón: 0009-0000-3837-7151

I. Rausch: 0000-0002-4007-1669

C. la Fougère: 0000-0001-7519-0417

F. P. Schmidt: 0000-0003-1886-4803


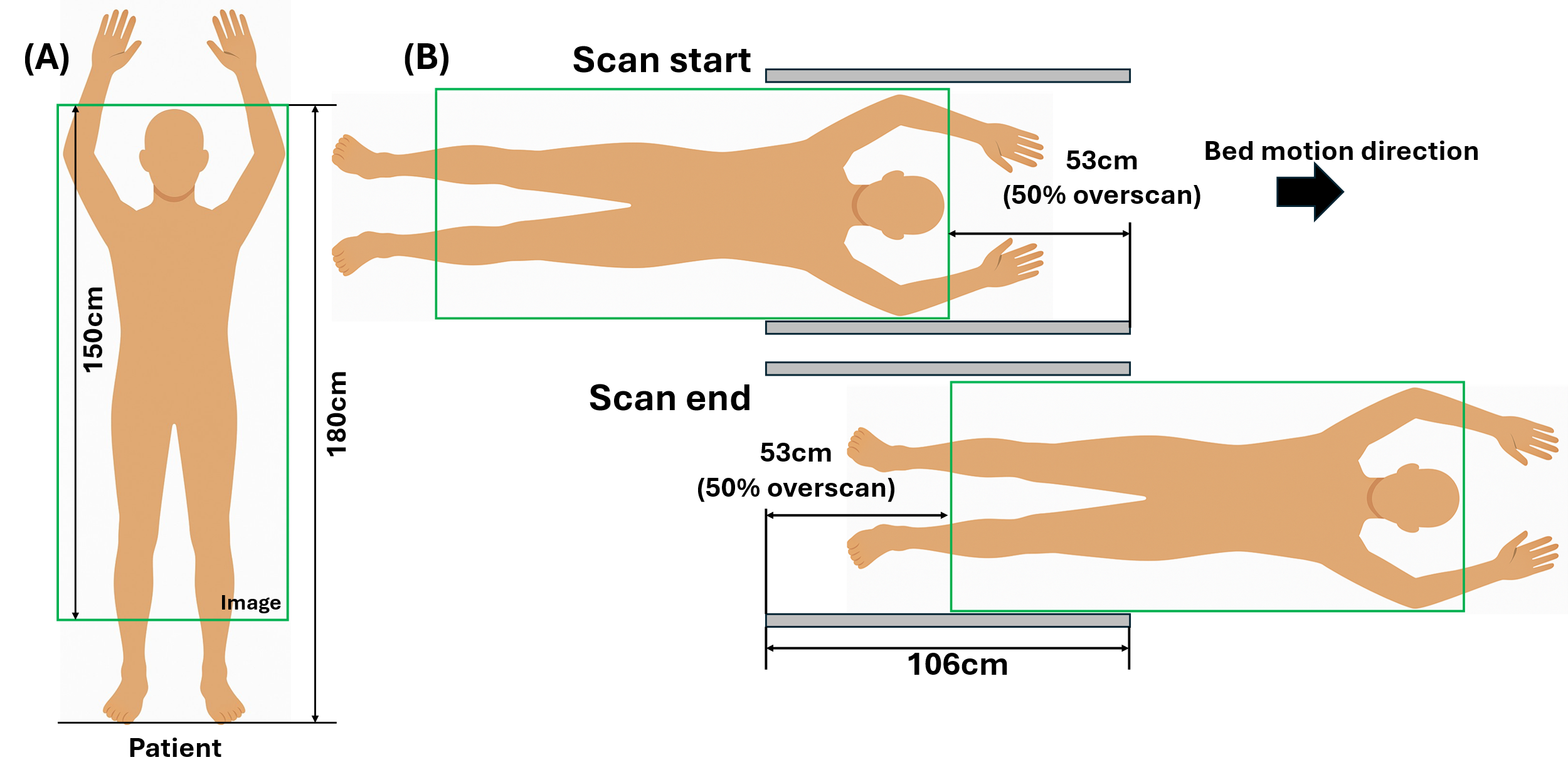


**Supplementary Figure 1** (A) Illustration of a patient with 180 cm height and an intended axial image coverage of 150 cm (green box). (B) Illustration of the start and end positions during a 150 cm CBM acquisition with 50% overscan on the Biograph Vision Quadra PET/CT scanner


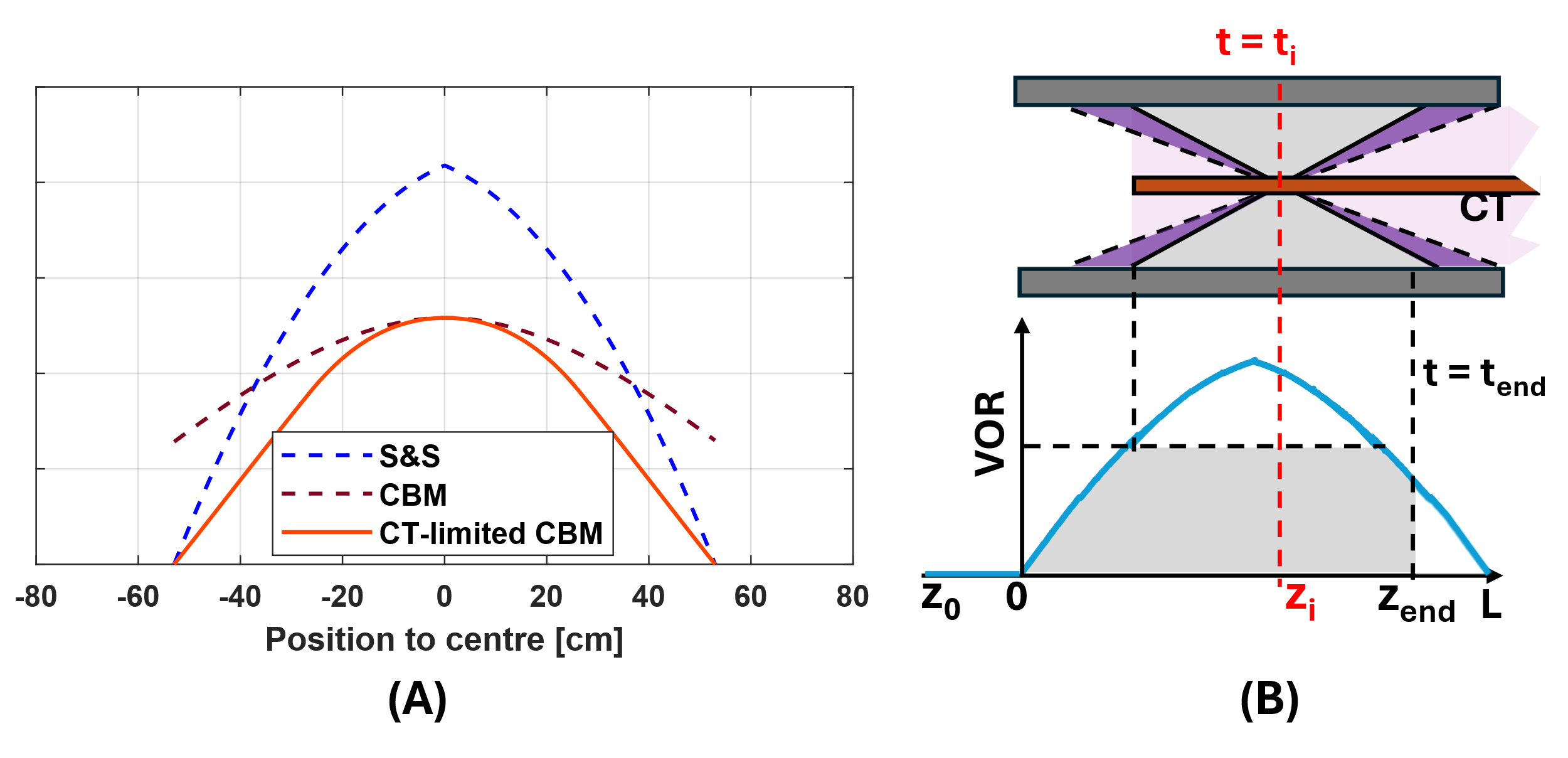


**Supplementary Figure 2** (A) Illustration of theoretical axial count profiles in CBM acquisitions on the Biograph Vision Quadra, with and without CT range limitations; (B) Schematic representation of the impact of limited acceptance angle to preserve attenuation correction with limited CT range in CBM on VOR, highlighting preserved lines of response (LORs) within the CT range (grey area) and discarded LORs outside the CT coverage (purple area)


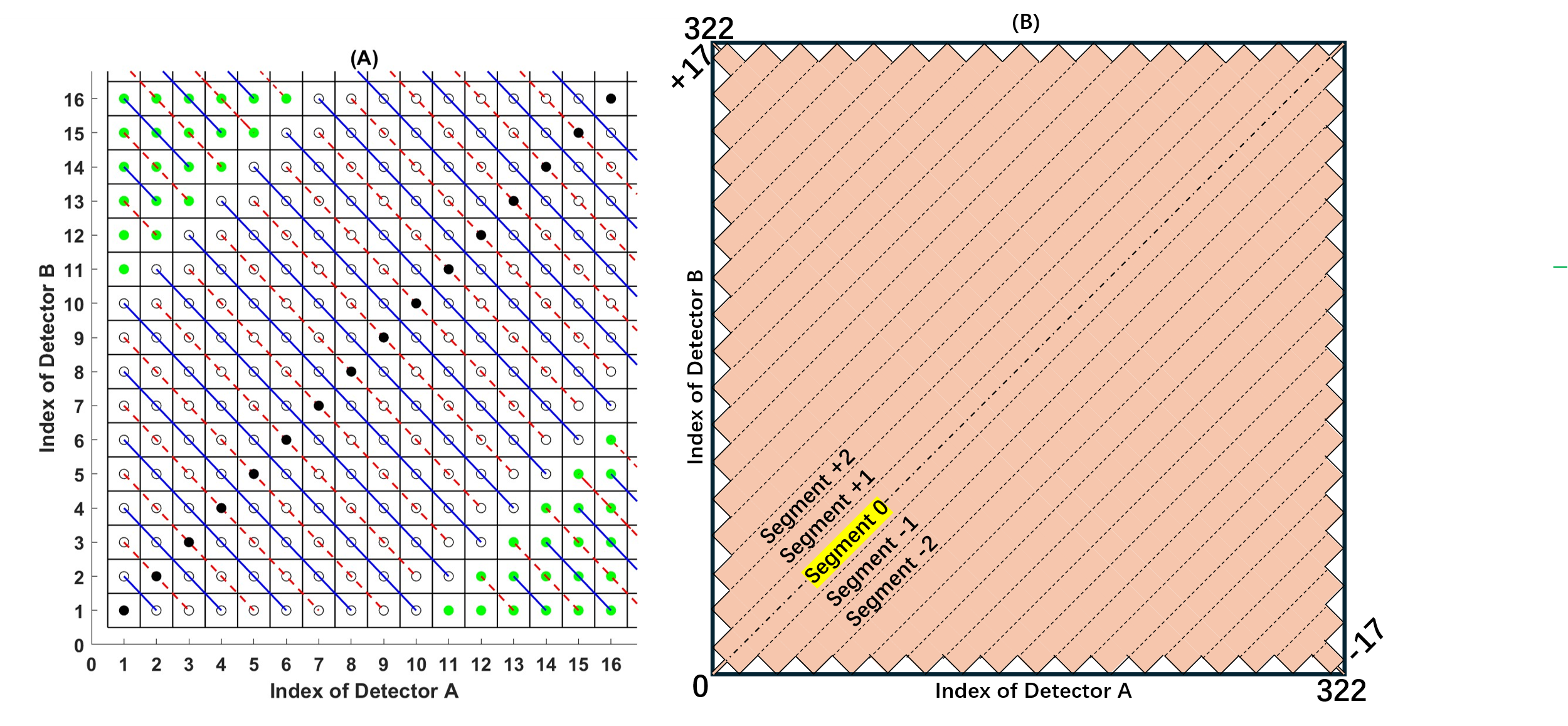


**Supplementary Figure 3** (A) Michelogram of the Biograph Vision Quadra in S&S mode with span 19, exemplarily shown for detector indices 1 to 16. In S&S, the LORs contributing to image reconstruction increase progressively at the axial edges, e.g., here for segment 0, LORs for detectors 1 to 10 gradually expand until the complete span of 19 is reached. In CBM, however, edge slices are formed from complete span 19 segments, resulting in the schematic Michelogram (B) with its saw-tooth pattern, which gives rise to the staircase pattern observed in the axial count profiles (Fig. 6)

**Supplementary Table 1**: Overview of the sequential S&S and CBM measurement protocols for the tube and IEC phantom experiments. A 380 s S&S acquisition served as the reference and was followed by CBM scans with bed speeds of 2.8–50 mm/s for the tube phantom and 2.8 and 8.4 mm/s for the IEC phantom. For each dataset, the time delay relative to the reference start time, the measured activity concentration with its relative deviation from the reference, and the corresponding decay correction factor are reported.

| Phantom/position | Datasets | Time delay with respect to reference start time | Activity concentration [kBq/mL] and relative deviation from reference [%] | Decay correction factor |
| --- | --- | --- | --- | --- |
| Tube phantom | S&S (reference) | Reference | 2.20 | 1 |
|  | CBM (2.8 mm/s) | 6 min 40 sec | 2.11 (-4.1%) | 1.04 |
|  | CBM (4.8 mm/s) | 13 min 13 sec | 2.02 (-8.1 %) | 1.09 |
|  | CBM (8.4 mm/s) | 17 min 13 sec | 1.97 (-10.5 %) | 1.11 |
|  | CBM (50 mm/s) | 19 min 33 sec | 1.94 (-11.8 %) | 1.13 |
| IEC phantom  (0 cm offset) | S&S (reference) | Reference | 2.10 | - |
|  | CBM (2.8 mm/s) | 6 min 33 sec | 2.01 (- 4.3%) | - |
|  | CBM (8.4 mm/s) | 16 min 57 sec | 1.89 (-10.0 %) | - |
| IEC phantom  (45 cm offset) | S&S (reference) | Reference | 1.84 | - |
|  | CBM (2.8 mm/s) | 6 min 35 sec | 1.76 (-4.3 %) | - |
|  | CBM (8.4 mm/s) | 17 min 3 sec | 1.65 (-10.3 %) | - |
| IEC phantom  (50.5 cm offset) | S&S (reference) | Reference | 1.61 | - |
|  | CBM (2.8 mm/s) | 6 min 33 sec | 1.54 (-4.3 %) | - |
|  | CBM (8.4 mm/s) | 16 min 59 sec | 1.44 (-10.5 %) | - |
